# Beyond the Critical Threshold: Elastic Fiber Remodeling and Fracture in the Pathogenesis of Pulmonary Emphysema

**DOI:** 10.3390/ijms262210930

**Published:** 2025-11-12

**Authors:** Jerome Cantor

**Affiliations:** School of Pharmacy and Allied Health Sciences, St. John’s University, Queens, NY 11439, USA; cantorj@stjohns.edu

**Keywords:** elastin, elastic fibers, desmosine, crosslinking, pulmonary emphysema

## Abstract

Pulmonary emphysema is a progressive and debilitating lung disease characterized by the destruction of alveolar walls and enlargement of airspaces, resulting in impaired gas exchange and reduced lung function. Central to this pathology is the degradation of the extracellular matrix (ECM), particularly the elastic fiber network containing elastin protein responsible for storing and releasing the energy that expels air from the lung. Both intrinsic and extrinsic mechanical stress play a pivotal role in ECM remodeling, influencing elastin degradation and the structural integrity of alveolar walls. This paper explores the interactions between mechanical forces and ECM components, emphasizing the role of increased elastin crosslinking in the pathogenesis and progression of emphysema. The molecular mechanisms responsible for this process are described in the context of emergent phenomena associated with alveolar wall distension and rupture, including the role of diagnostic biomarkers in the early detection of elastic fiber injury that may facilitate timely therapeutic interventions designed to preserve ECM integrity and improve patient outcomes.

## 1. Introduction

Pulmonary emphysema, a major component of chronic obstructive pulmonary disease (COPD), involves irreversible destruction of alveolar walls and progressive airspace enlargement. The disease is driven by a complex interplay of inflammatory, enzymatic, and mechanical factors that disrupt the structural integrity of the lung parenchyma. Increased mechanical stress can cause extracellular matrix (ECM) degradation, particularly when accompanied by an imbalance between protease and antiprotease activity [1,2]. Enhanced activity of matrix-degrading enzymes, such as neutrophil elastase and matrix metalloproteinases (MMPs), adversely affects ECM structural integrity, resulting in alveolar wall distention and rupture, reduced surface area for gas exchange, and compromised mechanical properties of the lung [3,4].

This process is exacerbated by lung mechanical stress arising from transpulmonary pressure during breathing, coughing, airway obstruction, and hyperinflation of alveoli due to air trapping [5,6]. A complex network of elastic fibers in alveolar walls normally absorbs and redistributes these forces. However, the degradation of elastin, the distensible elastin component of these fibers, disrupts this balance and leads to focal concentration of stress and tissue rupture [7]. The resulting micro-injuries in alveolar walls induce an inflammatory response accompanied by ECM degradation. Three dimensional computational models of pulmonary emphysema have shown that stress concentrations are highest at the junctions where the alveolar septa meet, making these regions particularly susceptible to damage [8]. Other lung areas have more balanced stress distribution and are less subject to mechanical deformations that contribute to elastic fiber fragmentation. Furthermore, the alveoli lining the alveolar ducts are less susceptible to rupture than those at the tips of the ducts because they are more tethered to neighboring alveoli and connective tissue, which helps distribute mechanical forces more evenly and reduces localized stress.

The interplay between enzymatic degradation and mechanical stress accelerates the breakdown of elastin, creating a vicious cycle of tissue destruction [3,9]. This damage may involve initial increases in elastin crosslinking that enhance the structural stability of elastic fibers. However, once alveolar wall diameter exceeds a threshold value, the balance between elastic fiber injury and repair undergoes a phase transition involving rapid elastin breakdown and irreversible tissue damage [10]. The subsequent acceleration of airspace enlargement emphasizes the importance of early detection of alveolar wall injury that facilitates timely therapeutic intervention.

**Figure 1:**
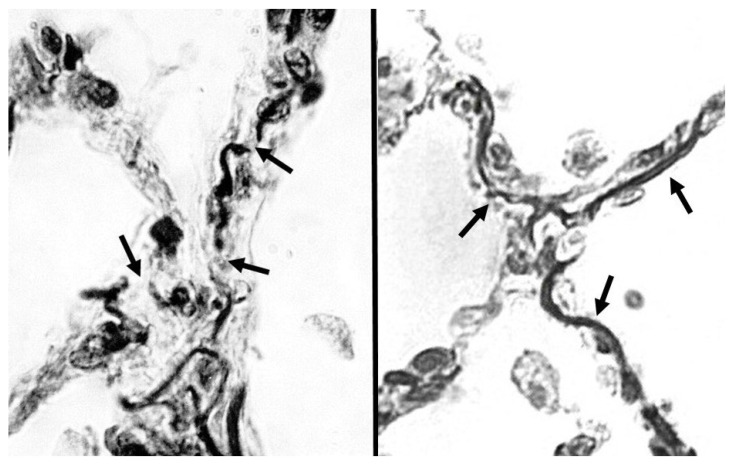
(**Left**) Human postmortem emphysematous lung showing fragmented elastic fibers (arrows) in alveolar walls. (**Right**) Normal human postmortem lung with intact elastic fibers (arrows). Orcein stain; 1000× magnification. Left panel reprinted with permission [10].

The nonlinear progression of disease may be modeled using percolation theory, where fluid movement through a network of interconnected channels represents the progression of chemical and physical phenomena [11]. Regarding the lung, the percolation of mechanical forces through the elastic fiber network represents an analogous process, in which the fragmentation of elastic fibers resembles the random disconnection of percolation bonds (Figure 1). A phase transition occurs when a critical threshold is reached, involving a widespread loss of connectivity that results in uneven transmission of mechanical forces and the emergence of clinically apparent disease [12]. This transition may be manifest by changes in lung compliance and gas exchange.

The fragmentation and unraveling of elastic fibers that accompany the phase transition is characterized by a biphasic response involving an initial increase in elastin crosslinking, followed by an accelerating breakdown of these fibers and an exponential increase in the release of crosslinks from disintegrating elastin peptides [10]. This phase transition remains poorly understood due to the difficulty correlating biochemical changes in elastin with morphometric measurements of alveolar wall distention. In the current paper, we use previously reported mass spectrometric measurements of the unique elastin crosslinks, desmosine and isodesmosine (DID), in normal and emphysematous human postmortem lungs to construct a proposed mechanism for a phase transition based on changes in elastic fiber mechanical properties.

## 2. Experimental Findings

This laboratory performed measurements of peptide-free DID in the lungs of hamsters with pulmonary emphysema induced by treatment with both cigarette smoke and lipopolysaccharide [13]. The results indicated a significant correlation between the level of free lung DID and alveolar diameter, supporting the role of elastin crosslinks as a biomarker for airspace enlargement.

**Figure 2:**
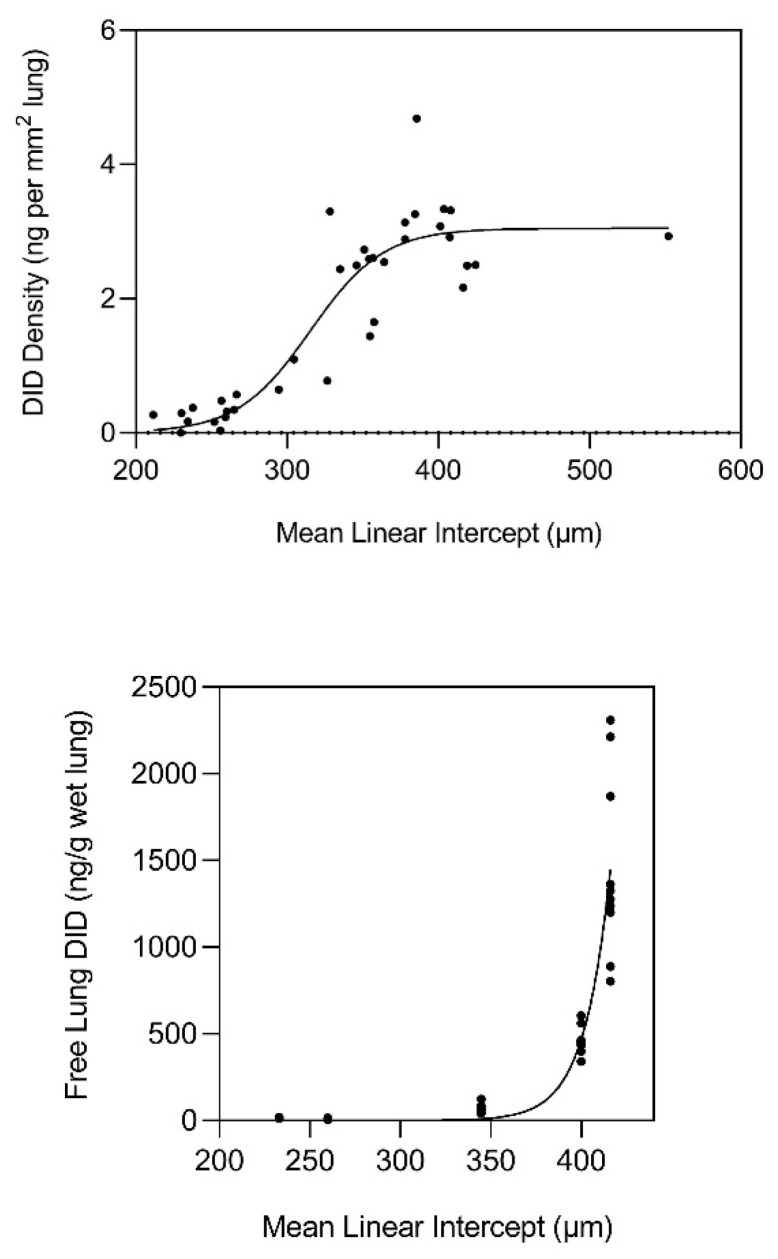
(**Upper**) The content of free lung DID in human postmortem lungs increases exponentially when alveolar diameter exceeds 400 µm, suggesting that this value is the fracture threshold for elastin. (**Lower**) The density of DID in normal and emphysematous human postmortem lungs increases exponentially when airspace size exceeds 300 µm and levels off at 400 µm. Both panels reprinted with permission. Reprinted with permission [10].

This study was followed by measurements of free lung DID in multiple random distal lung samples from normal and emphysematous postmortem human lungs, which showed that elastin breakdown was greatly accelerated when the mean airspace diameter exceeded 400 μm (Figure 2). The density of DID in tissue sections free of large airways and major blood vessels also increased markedly beyond 300 μm and leveled off at 400 μm (Figure 2). In both cases, the correlation between DID and alveolar diameter was unaffected by differences in comorbidities.

**Figure 3:**
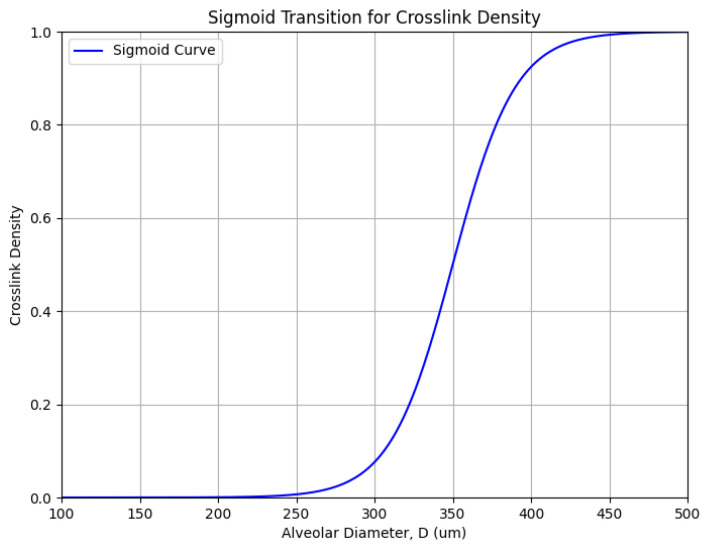
Sigmoid curve representing the mathematical relationship between crosslink density and alveolar diameter. The critical value (Dc) for the exponential increase in density is 300 µm, based on the experimental data in the upper panel of Figure 2.

The effect of alveolar wall distention on crosslinking density may be modeled as a sigmoid curve (Figure 3), where DID density [σ(x)] undergoes an exponential increase (expressed as values between 0 and 1) when alveolar diameter (D) reaches a critical value (Dc) of 300 µm, based on the experimental findings (Figure 2, upper panel) [10]. The addition of this empirical constant provides a means of tuning the following equation to the physical events associated with injury and repair of elastic fibers:

σ(x) = 1/1 + e^−k(D−Dc)^

Here, e represents Euler’s number (~2.718), a fundamental constant used in exponential functions that describe continuous growth or decay, and k is an empirical constant that determines the rate of increase in crosslink density, based on the data shown in the upper panel of Figure 2.

**Figure 4:**
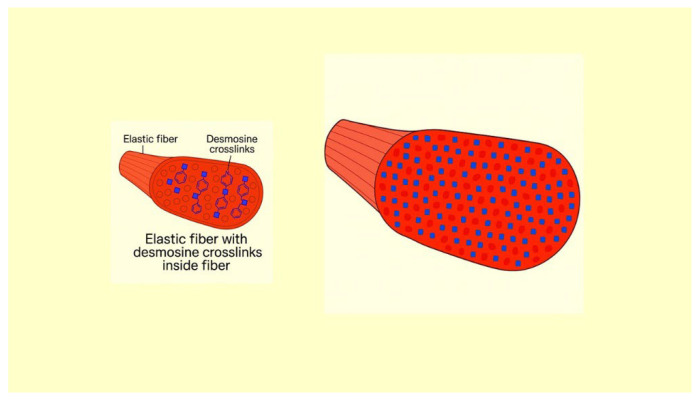
Illustration showing the increase in DID density in elastic fibers due to the synthesis of elastin peptides during the repair process.

This relationship is supported by in vitro studies demonstrating that mechanical stretching enhances the formation of elastin crosslinks (Figure 4). When subjected to tensile forces, elastin fibers undergo conformational changes that promote the interactions necessary for crosslinking [14]. Additionally, mechanical stretching can increase the expression of lysyl oxidase (LOX), an enzyme that facilitates the crosslinking of elastin molecules [15].

These results suggest that the initial stages of alveolar wall injury are characterized by a balance between elastic fiber injury and repair in which greater crosslink density reflects enhanced elastin synthesis. However, when alveolar diameter exceeds 400 μm, the repair process undergoes a decompensatory phase reflected by a marked release of free DID and fragmentation of elastic fibers [10]. While the enhanced crosslinking initially prevents elastic fiber unraveling and fragmentation, it subsequently evolves into a pathological process involving mechanically induced rupture of increasingly rigidified fibers.

The acceleration of elastin breakdown with increasing alveolar wall distention is consistent with the hypothesis that pulmonary emphysema is an emergent phenomenon involving a phase transition to an active disease state that may be more resistant to therapeutic intervention. This transition may involve the loss of elastic fiber structural integrity as the effects of increasing alveolar wall strain cause the excessively crosslinked elastin to undergo fracture.

**Figure 5:**
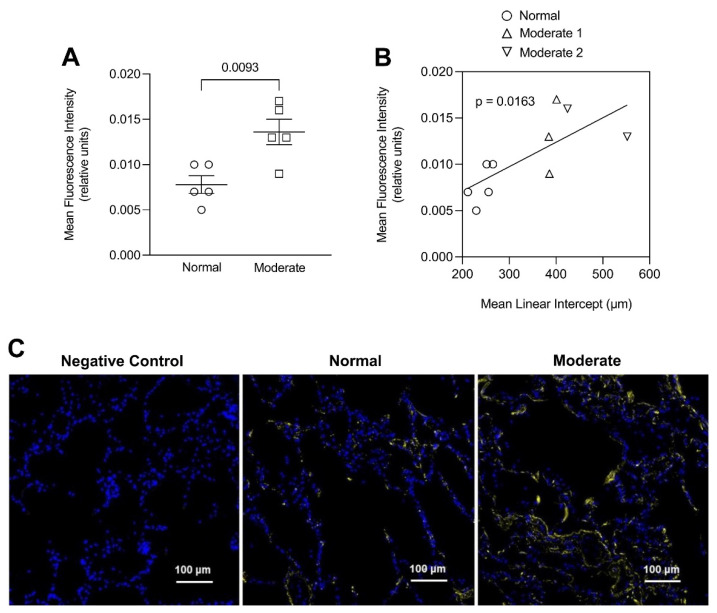
(**A**) Elastin immunofluorescent staining was significantly increased in human postmortem lungs with moderate emphysema compared to. (**B**) The staining intensity significantly correlated with the mean linear intercept, a measure of airspace size. (**C**) Photomicrographs of elastin immunostaining in the alveolar walls of normal and moderately emphysematous human postmortem lungs. The staining pattern in the diseased lung reflects an increase in elastin associated with elastic fibers. Reprinted with permission [10].

Although previous investigations have shown that pulmonary emphysema is associated with either similar or decreased elastic fiber content compared with normal lungs, our results suggest that earlier stages of the disease may involve a more balanced relationship between injury and repair, where the damaging effects of inflammation and alveolar wall strain are offset by increased elastin crosslinking [16]. While there are few remaining lysine residues for crosslinking in mature elastic fibers, additional DID may result from the conversion of bifunctional crosslinks (e.g., lysinonorleucine) to DID [17]. Alternatively, increased crosslink density may involve enhanced synthesis of elastin peptides incorporated into existing elastic fibers during the repair process [18]. This mechanism is supported by immunofluorescence studies showing a significant correlation between alveolar wall elastin content and airspace size (Figure 5) [10].

**Figure 6:**
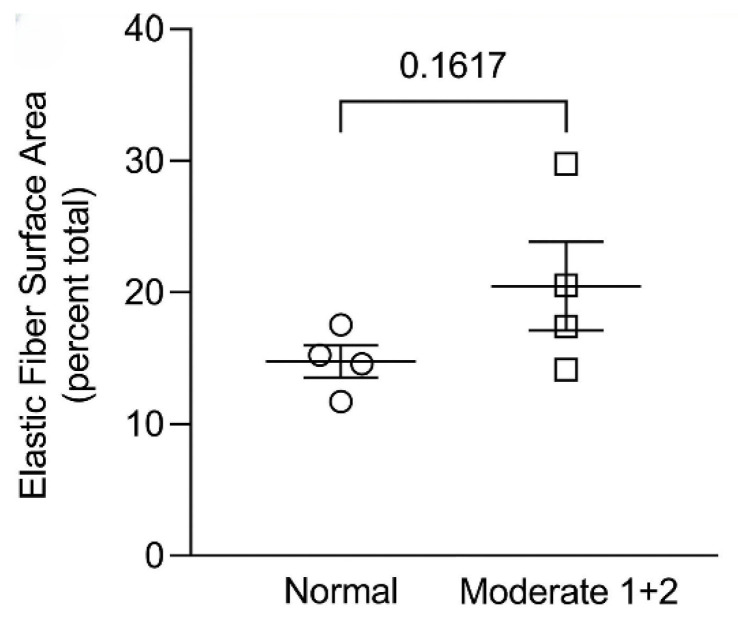
The surface area of elastic fibers in a human postmortem lung with moderate emphysema was not increased compared to normal, whereas the immunofluorescence studies show a significant increase in elastin content (Figure 5). This finding is consistent with preferential resynthesis of elastin rather than the entire elastic fiber. Reprinted with permission [10].

In contrast, alveolar wall elastic fiber surface area was not significantly increased, suggesting that the repair process mainly involves resynthesis of elastin rather than the entire fiber (Figure 6) [10]. Other studies support this concept by showing that de novo formation of intact elastic fibers in pulmonary emphysema is largely ineffective [19].

## 3. Fragmentation of Hypercrosslinked Elastic Fibers

### 3.1. Elastin Rigidity Due to Hypercrosslinking

It is hypothesized that the fragmentation of elastic fibers results from the continued increase in elastin rigidity, where the elastic modulus of a crosslinked polymer is directly proportional to the crosslink density [20,21]. A common formula used to describe this relationship is:

E = 3ρRT/Mc

where the elastic modulus (E) is directly proportional to the polymer density (ρ), the ideal gas constant (R), and the absolute temperature (T), and inversely proportional to the molecular weight between crosslinks (Mc). A higher density usually means more polymer chains packed together, potentially leading to more crosslinks and a higher elastic modulus [20].

**Figure 7:**
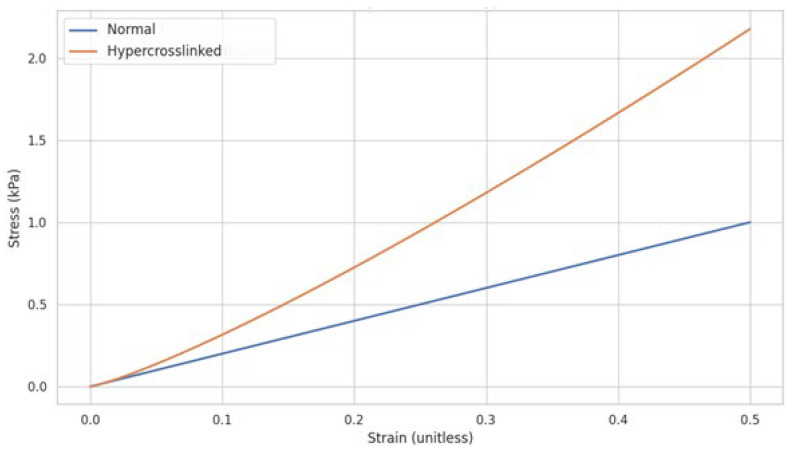
Graphic illustration of the changes in elastic fiber response to mechanical strain based on crosslink density.

Limitations to this equation include the possibility that not all crosslinks are equally effective in contributing to the elastic modulus. Some may be located in regions where elastic fibers have undergone injury are therefore less effective in restricting chain movement [22]. Nevertheless, once a critical threshold is reached, these fibers begin to rigidify, and its shear modulus grows with a power-law dependence on the crosslink density (Figure 7).

### 3.2. Biphasic Elastin Fracture Curve

**Figure 8:**
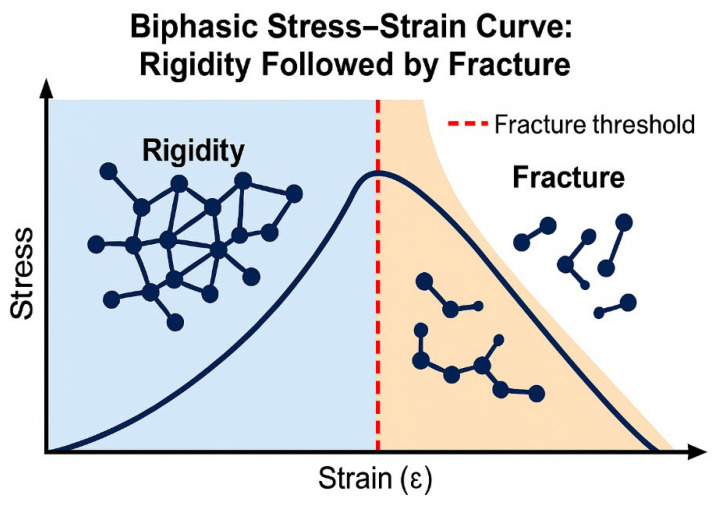
Graphic illustration of the initial increase in elastic fiber rigidity due to hypercrosslinking of elastin, followed by fracture and degradation of the fibers.

As elastin rigidity increases due to excessive crosslinking, it initially becomes more resistant to fracture. However, the continuation of this process results in a transition to a more rigid state, which increases the probability of fracture as alveolar wall strain intensifies (Figure 8) [23,24].

The biphasic relationship based on increasing elastin crosslink density may be expressed as follows:

F = aX − bX^2^

where F represents the fracture resistance or strength of elastin, X represents the crosslink density, a is a constant that defines the shape of the pre-fracture curve, and b is a constant that determines how quickly the post-fracture curve falls off.

**Figure 9:**
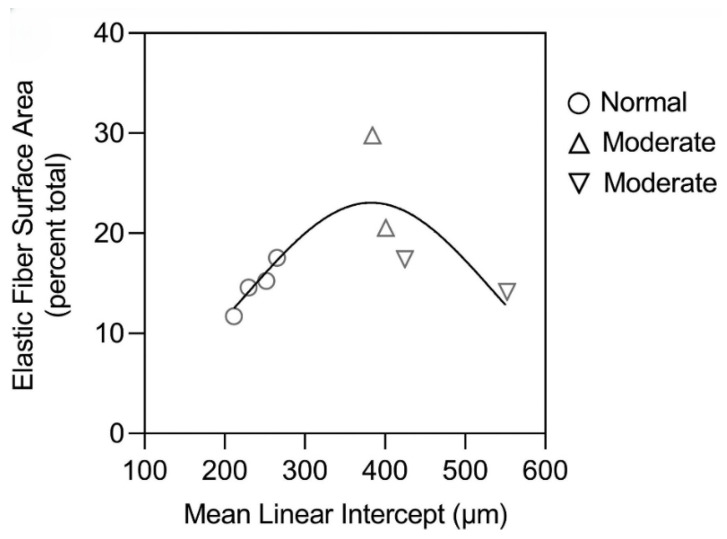
The biphasic curve correlating elastic fiber surface area with airspace enlargement is similar to the one in Figure 8 describing stress-induced fracture, suggesting that fragmentation of these fibers increases their susceptibility to degradation. Reprinted with permission [10].

The similarity of the curve shown in Figure 8 to one correlating elastic fiber surface area with airspace size in normal and emphysematous human postmortem lungs suggests that fragmentation of elastic fibers increases their susceptibility to enzymatic and oxidative degradation (Figure 9).

## 4. The Role of Hydration in Elastic Fiber Fracture

Another component that may play a significant role in lung mechanics is interstitial water content. The interaction of hydrophobic groups in elastin with adjacent water molecules produces a positive free energy change that contributes to the storage of elastic energy [25]. The retention of water also facilitates the swelling of the elastin molecules, increasing their random energy state and enhancing recoil due to greater entropy loss during distention. Consequently, decreased water availability can adversely affect the elasticity of alveolar walls.

The mechanical properties of elastic fibers may also depend on the intramolecular forces of attraction among elastin peptide chains [26]. Removing water molecules reduces the distance between the elastin peptide chains, enhancing their cohesion and impairing the distensibility of the fibers [27]. This process is likely to occur in pulmonary emphysema, where decreased blood flow and damage to the hydrophilic components of the ECM result in a loss of interstitial water. This concept is supported by studies showing that human emphysematous lungs have a significant decrease in hyaluronan (HA), a long-chain, hydrophilic polysaccharide in proximity to elastic fibers [28]. The removal of HA would reduce the elasticity of elastic fibers, increasing their susceptibility to fracture.

## 5. DID as a Biomarker of Therapeutic Efficacy

The accelerated release of free DID when airspace size exceeded 400 µm is consistent with the fragmentation of elastic fibers, where mechanical stress exceeds the fracture threshold for elastin [10]. Conversely, a decline in tissue and fluid levels of DID following therapeutic intervention may indicate a shift in the remodeling of elastic fibers toward a more stable configuration, reducing the risk of alveolar wall rupture.

Currently, the only recognized clinical trial endpoints for evaluating potential treatments for pulmonary emphysema are pulmonary function studies, which lack sensitivity and may not accurately measure therapeutic efficacy [29]. High-resolution CT is a more sensitive alternative but may require an extended period to demonstrate the preservation of lung mass [30,31]. While various inflammatory mediators have also been proposed as therapeutic biomarkers, the level of DID crosslinks may have greater specificity for airspace enlargement disease because they are better indicators of structural changes in alveolar walls [32,33].

While concurrent elastic fiber injury in blood vessels and other tissues could adversely affect the specificity of the DID biomarker, it may nevertheless serve as a real-time indicator of therapeutic efficacy in clinical trials of novel treatment agents for pulmonary emphysema. A significant difference in crosslink levels between closely matched experimental and control groups would offset confounding factors and provide strong evidence of a positive treatment effect. Using sputum and possibly breath condensate to measure free DID would also increase the specificity for pulmonary emphysema.

To determine the role of DID levels in evaluating drug efficacy, our laboratory incorporated this biomarker in a 28-day clinical trial of aerosolized HA in patients with alpha-1 antiprotease deficiency-induced pulmonary emphysema [34]. Inhalation of HA twice daily significantly decreased the amount of free DID in urine over the course of the trial, whereas levels in the placebo group remained unchanged. Free urinary DID was a more sensitive indicator of a treatment effect than total DID in either urine or plasma. This finding may be explained by the extensive release of free DID in the human postmortem emphysematous lungs (Figure 2, lower panel), which is rapidly excreted into the urine.

## 6. Therapeutic Strategies Targeting Elastin Preservation

### 6.1. Matrix Replacement Therapy

The clinical trial of aerosolized HA evolved from a series of translational studies that began with the observation that pretreatment with hyaluronidase increases airspace enlargement in a pulmonary emphysema model induced by intratracheal elastase instillation [35]. Subsequently, it was shown that pretreatment with HA significantly reduced airspace enlargement in emphysema models induced by elastase or cigarette smoke [35].

**Figure 10:**
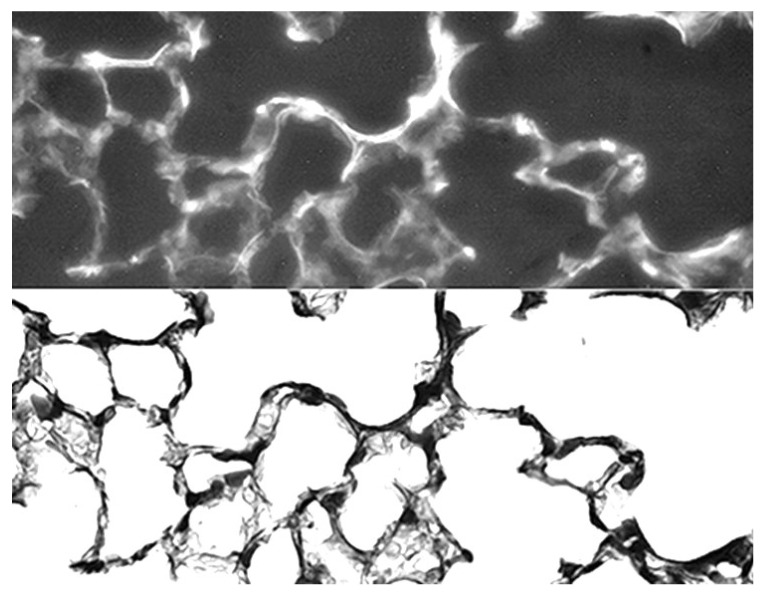
Photomicrographs of mouse lung at 24 h following intratracheal instillation of fluorescein-labeled HA. Prominent fluorescence associated with elastic fibers (**upper**) was confirmed by a photomicrograph of the same area (**lower**) showing an identical pattern of elastic fiber staining. Reprinted with permission. Original magnification: 400×. Reprinted with permission [35].

This protective effect involved the binding of HA to elastic fibers, where it functions as a physical barrier preventing the degradation of elastic fibers by various agents, including neutrophil elastase and MMPs (Figure 10). The attachment of HA to elastic fibers may involve the formation of electrostatic or hydrogen bonds. located on either the elastin protein itself or the surrounding microfibrillar component. Due to its self-aggregating properties, the inhaled HA may form much larger complexes that more effectively protect elastic fibers against elastases and the cells that secrete them [36].

HA may also prevent alveolar wall rupture by improving the mechanical properties of elastin. Negatively charged carboxyl groups within the molecule expand its domain and facilitate the entrapment of water in the proximity to elastin [37]. The interaction between water molecules and hydrophobic domains of elastin produces a positive change in free energy that expels air from the lung, preventing the distention and rupture of alveolar walls. While the actual effect of HA on pulmonary mechanics remains to be determined, preclinical testing by an independent laboratory suggested that it may improve lung hydration. Rats exposed to nebulized HA for two weeks showed a dose-dependent increase in lung weight, which was not due to cellular proliferation (Inveresk Laboratories report no. 20954, 2002). Microscopically, no evidence of pulmonary edema was seen, consistent with the absorption of water by the extracellular matrix.

### 6.2. Crosslink Inhibition

Targeting LOX with small molecule inhibitors represents another therapeutic strategy to mitigate pathological crosslinking. An example of this approach is the treatment of experimentally induced pulmonary fibrosis with β-aminopropionitrile (BAPN), an inhibitor of LOX. Decreasing the activity of this enzyme lowers crosslink density and modifies the biomechanical properties of the affected tissues [38].

In preclinical models of fibrosis, such as those involving lung, liver, or kidney tissues, administration of BAPN has demonstrated significant efficacy. In a murine model of bleomycin-induced pulmonary fibrosis, BAPN treatment resulted in decreased collagen deposition as evidenced by histological staining for collagen [38]. Additionally, biochemical assays revealed a marked decline in the levels of soluble collagen and hydroxyproline, further supporting the notion that BAPN-induced LOX inhibition leads to alterations in ECM deposition [39]. However, the toxicity of this agent may preclude its administration to humans, necessitating the use of other crosslink inhibitors such as penicillamine, which has a safety profile compatible with clinical trials [40].

Whether the dose of LOX inhibitors can be titrated to reduce elastin crosslinking without weakening alveolar wall structural integrity and promoting airspace enlargement remains to be seen. Optimizing the dosing regimen, evaluating the long-term safety of these agents, and exploring combination therapies with other antifibrotic agents will be crucial steps in translating these findings into an effective treatment for pulmonary emphysema. Additionally, developing more selective small-molecule inhibitors that specifically target LOX could minimize potential side effects.

### 6.3. Inhibition of Elastin-Derived Peptides

Elastin-derived peptides (EDPs) contain specific amino acid sequences that can bind to specialized receptor complexes that induce the synthesis of proinflammatory cytokines. The use of elastase inhibitors to prevent elastin degradation and limit the formation of these peptides has been the focus of a number of clinical investigations. One recent trial examined the therapeutic efficacy of an oral neutrophil elastase inhibitor in patients with alpha-1 antiprotease deficiency [41]. Measurement of serum DID levels showed a significant reduction in the treatment group compared to the placebo. However, the alpha-1 antiprotease-deficient population only represents a small subset of patients with a specific increase in neutrophil elastase, whereas the broader disease process involves multiple elastases.

Inhibiting the activity of peptidyl arginine deiminase (PAD) may also provide a means of decreasing the production of EDPs. This enzyme was shown to increase the susceptibility of elastin to degradation in vitro, and treatment of LPS-induced pulmonary emphysema with PAD decreased lung elastance and exacerbated airspace enlargement [42,43]. Conversely, the use of a PAD inhibitor in the PAD/LPS model reversed these findings, suggesting that inhibition of PAD could potentially provide a therapeutic option to reduce the breakdown of elastin and limit the formation of EDPs [43].

An alternative therapeutic approach involves blocking the interaction between EDPs and the elastin receptor complex (ERC), which may mitigate inflammation and elastic fiber injury [44]. The release of EDPs during elastin degradation promotes vascular inflammation and stiffness due to their ability to activate various signaling pathways in vascular smooth muscle cells and endothelial cells [7]. This process leads to a cascade of events, including the release of proinflammatory cytokines and increased MMP activity.

Recent studies have focused on developing neuraminidase 1 (NEU-1) inhibitors and ERC antagonists as therapeutic agents that preserve vascular elasticity and prevent age-associated arterial stiffness [45]. NEU1 is an enzyme responsible for the desialylation of glycoproteins and glycolipids, a process thought to enhance the bioavailability of EDPs by exposing underlying receptor sites on cells [46]. By inhibiting NEU1, it is hypothesized that the resulting reduction in EDP activity would limit the production of proinflammatory molecules responsible for the degradation and remodeling of elastic fibers.

Another strategy involves the use of ERC antagonists that prevent their interaction with EDPs, thereby blocking downstream signaling pathways that promote inflammation and remodeling. Recently developed ERC antagonists have shown efficacy in reducing EDP-induced vascular changes associated with elastic fiber remodeling in arterial walls [47].

Furthermore, studies suggest a synergistic effect of using NEU1 inhibitors alongside ERC antagonists, potentially amplifying their benefits in reducing inflammation and ECM deposition. However, this therapeutic strategy’s long-term efficacy and safety profile need to be determined before translating to the clinical setting.

### 6.4. Enhancing Tropoelastin Synthesis

While ventilatory forces may preclude reconnection of ruptured alveolar walls, it may be possible to regenerate the elasticity of intact septa by inducing the resynthesis of normally crosslinked elastic fibers. This process would require the restoration of the biochemical mechanisms responsible for the initial formation of these fibers in the developing lung, including the interaction of tropoelastin with other matrix components. Strategies to upregulate tropoelastin expression may involve using several agents that promote elastin precursor synthesis and enhance matrix assembly in engineered tissues [48]. Regarding this possibility, insulin-like growth factor 1 (IGF-1) and transforming growth factor beta 1 (TGF-β1) have significantly increased elastin synthesis [49].

Studies indicate that IGF-1 enhances tropoelastin expression in cultured fibroblasts following treatment with this agent [50]. Elevated levels of tropoelastin protein were also seen, indicating that IGF-1 stimulates transcription and enhances translation of elastin precursors. Similarly, TGF-β1 has been shown in vitro to increase tropoelastin production, confirming its effect on gene expression and protein synthesis [51]. These findings demonstrate the potential use of TGF-β1 and IGF-1 to promote elastin synthesis in alveolar walls at an early stage in the development of pulmonary emphysema, thereby preventing the mechanically induced distention that results in hypercrosslinking and fracture of elastic fibers.

In addition to using individual agents, biomimetic scaffolds and nanoparticles are being developed for the targeted delivery of multiple elastogenic molecules that support the regeneration of elastic fibers. Scaffolds combined with cytokines that promote elastogenesis significantly improved elastin deposition in vascular tissue, suggesting the possibility that inhaled biomaterials could preserve elastic fiber integrity and prevent alveolar wall injury [52].

With regard to therapeutically induced repair of elastic fibers, the DID biomarker could play an important role in monitoring its efficacy. As the mechanical properties of these fibers are reconstituted, their susceptibility to fracture and enzymatic degradation would be reduced, resulting in lower levels of free DID. However, further investigation of the correlation between elastic fiber restoration and levels of this biomarker is needed to determine the range of values consistent with a positive treatment effect.

## 7. Conclusions

Mechanical stress plays a central role in elastin degradation and remodeling of the ECM in pulmonary emphysema. The interplay between mechanical forces and biochemical processes leads to emergent phenomena that drive disease progression. While a variety of inflammatory mechanisms may play a role in the pathogenesis of pulmonary emphysema, the hypercrosslinking of elastic fibers and their subsequent fracture may be a universal process. Therapeutic strategies that preserve elastin integrity and modulate ECM remodeling could therefore alter the trajectory of the disease. This approach will require a holistic understanding of the mechanical and biochemical factors that contribute to airspace enlargement, including the emergent phenomena responsible for the loss of alveolar wall elasticity.

## Data Availability

No new data were created or analyzed in this study. Data sharing is not applicable to this article.
